# LoRA-DR-suite: adapted embeddings predict intrinsic and soft disorder from protein sequences

**DOI:** 10.1093/bioinformatics/btaf185

**Published:** 2025-07-15

**Authors:** Gianluca Lombardi, Beatriz Seoane, Alessandra Carbone

**Affiliations:** Sorbonne Université, CNRS, IBPS, Department of Computational, Quantitative and Synthetic Biology (CQSB), UMR7238, 4 Place Jussieu, Paris, 75005, France; Departamento de Física Téorica, Universidad Complutense de Madrid, Madrid 28040, Spain; Sorbonne Université, CNRS, IBPS, Department of Computational, Quantitative and Synthetic Biology (CQSB), UMR7238, 4 Place Jussieu, Paris, 75005, France; Institut Universitaire de France, Paris, France

## Abstract

Motivation. Intrinsic disorder regions (IDR) and soft disorder regions (SDR) provide crucial information on a protein structure to underpin its functioning, interaction with other molecules and assembly path. Circular dichroism experiments are used to identify intrinsic disorder residues, while SDRs are characterized using B-factors, missing residues, or a combination of both in alternative X-ray crystal structures of the same molecule. These flexible regions in proteins are particularly significant in diverse biological processes and are often implicated in pathological conditions. Accurate computational prediction of these disordered regions is thus essential for advancing protein research and understanding their functional implications. Results. LoRA-DR-suite addresses the challenge and employs a simple adapter-based architecture that utilizes protein language models embeddings as protein sequence representations, enabling the precise prediction of IDRs and SDRs directly from primary sequence data. Alongside the fast LoRA-DR-suite implementation, we release SoftDis, a unique soft disorder database constructed for approximately 500 000 PDB chains. SoftDis is designed to facilitate new research, testing, and applications on soft disorder, advancing the study of protein dynamics and interactions. Availability. LoRA-DR-suite and SoftDis database are available at https://huggingface.co/CQSB.

## 1 Introduction

While many proteins rely on a well-defined 3D structure to carry out their functions, a substantial fraction of the proteome in any organism consists of polypeptide segments that lack a stable and reproducible ordered structure (Dunker *et al.* 2000, Tompa 2002, Mészáros *et al.* 2009, Hu *et al.* 2018). Remarkably, these structurally flexible or amorphous regions often retain full functionality. Such unstructured and frequently underexplored segments are known as intrinsically disordered regions (IDRs). When the intrinsic disorder spans the entirety of a protein sequence, these proteins are classified as intrinsically disordered proteins.

IDRs are typically identified using structural techniques such as circular dichroism spectroscopy. In X-ray crystallography, they appear as missing residues or highly flexible regions, often indicated by elevated B-factor values (Sun *et al.* 2019). However, disorder is not always absolute—a residue may be absent in one structure yet resolved in another, reflecting a dynamic disorder-to-order transition. This inherent flexibility complicates the classification of disordered regions in protein structures. To address this challenge, the concept of *soft disorder* was introduced by Seoane and Carbone (2021), providing a unified framework to capture the ambiguity between ordered and disordered states. Large-scale statistical analyses of protein chains in the Protein Data Bank (PDB) revealed a strong correlation between soft disordered regions (SDRs) and protein interaction sites (Seoane and Carbone 2021). These regions appear to play a crucial role in the assembly and stabilization of protein complexes (Seoane and Carbone 2022). Beyond this, this study provided evidence of allosteric effects, where SDRs undergo spatial rearrangements upon binding, facilitating the formation of new interaction interfaces in larger, more complex assemblies.

Given their structural flexibility, IDRs and SDRs play essential roles in molecular recognition, signaling, and regulation and are implicated in numerous diseases (Iakoucheva *et al.* 2002, Dev *et al.* 2003, Melo *et al.* 2016, Uversky 2018). Accurately identifying and characterizing these regions is crucial for advancing protein research, therapeutic development by uncovering novel biological functions and deciphering complex molecular pathways (Dunker and Uversky 2010). A systematic exploration of IDRs and SDRs could offer critical insights into the mechanisms of poorly understood diseases and guide therapeutic strategies targeting disordered regions and their interaction networks.

Given the fundamental role of disordered regions, accurate predictive models are essential. Numerous computational tools identify IDRs, intrinsically disordered-binding regions, and flexible segments (Meng *et al.* 2017, Kurgan *et al.* 2023), with deep learning-based approaches increasingly dominant. To enhance their performance and practical applicability, recent efforts have systematically benchmarked these predictors using previously unresolved sequences, providing a robust assessment of their reliability and generalization capabilities (Necci *et al.* 2021, Del Conte *et al.* 2023a, 2023b). With the rapid production of genomic and metagenomic sequences, these predictive tools serve as a crucial link between sequence data and functional annotation, delivering structural insights without the cost, time, and limitations of experimental methods.

In this work, we present LoRA-DR-suite, a novel predictor for identifying IDRs and SDRs in protein sequences. The method leverages adapted embeddings and is built on an elegantly simple deep learning architecture.

## 2 Results

### 2.1 A suite of models based on adapted embeddings

**Figure 1.**
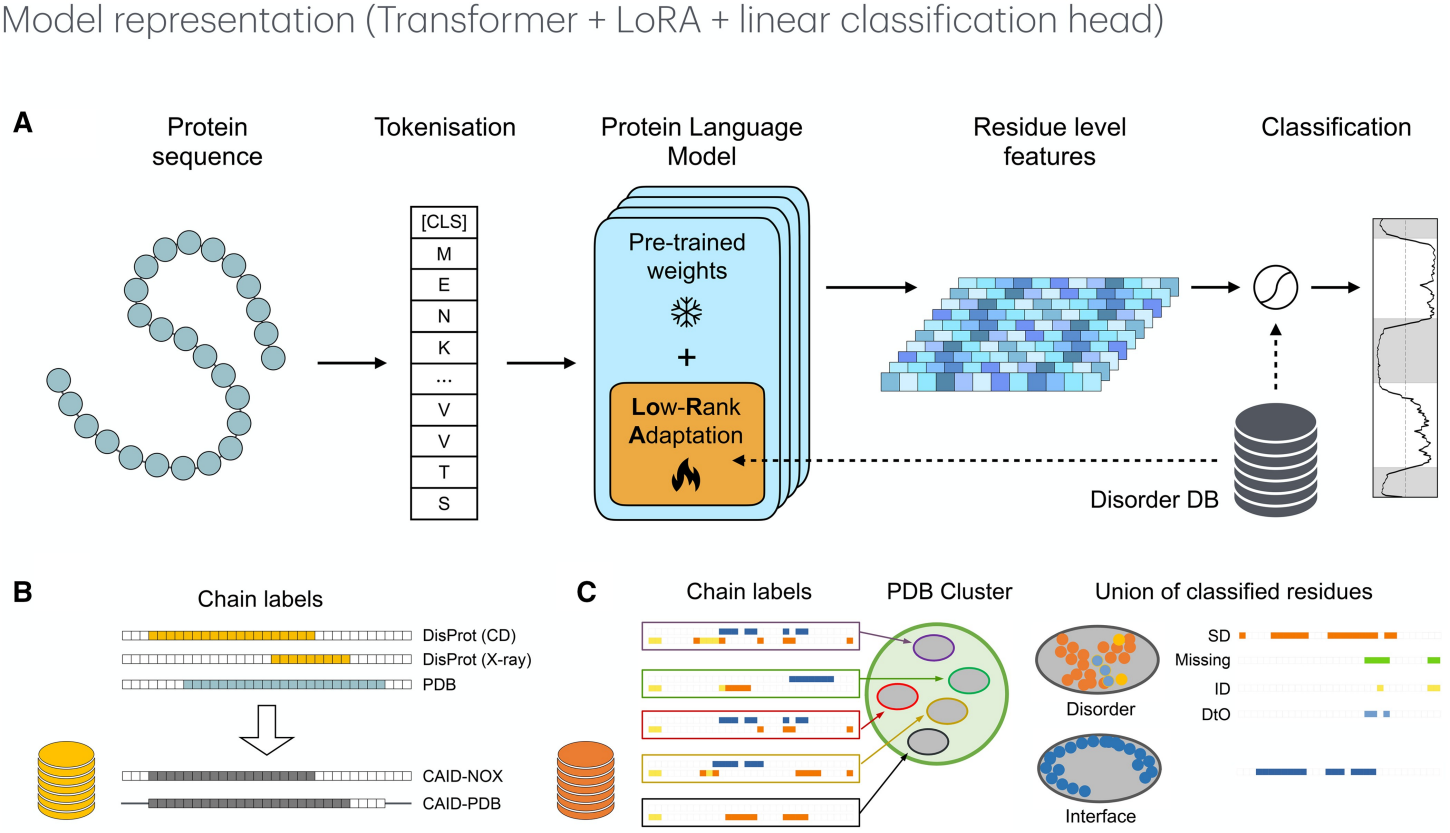
Model architecture and datasets construction. (A) Taking as input a protein sequence, after tokenisation, its adapted embedding is generated by a protein language model, with frozen weights, coupled with several LoRA layers. A linear classification head with dropout is used to generate a disorder profile for the protein, where scores of disorder are computed for each residue and used to construct a disorder profile for the entire sequence. (B) Three types of data are used to define intrinsic disorder. They are either coming from circular dichroism (CD) experiments, or X-ray crystallography (X-ray), or missing residues in a crystal (PDB). The CAID-PDB annotation is defined as the union of residues identified as intrinsically disordered by circular dichroism (CD) and X-ray crystallography, while the CAID-NOX annotation includes only residues identified by CD. Residues labeled as disordered or not by a method are represented by colored or white squares, respectively. Notably, missing residues in X-ray crystallographic data are unlabeled in the CAID-PDB annotation but assigned a negative label in the CAID-NOX annotation. (C) In the SoftDis database, soft disordered residues (SD), missing residues (Missing), intrinsically disordered residues (ID), and disorder-to-order transition residues (DtO) are identified by clusters of chains with a very similar protein sequence (see Materials and methods section). Each protein sequence associated to a PDB structure in a cluster (left) contributes information on missing residues (yellow squares), residues with high B-factor (orange square) and interface residues (blue squares). The union of these classified residues allows to label residues as SD, Missing, ID, DtO, and interface residues for the cluster and for its sequences (right).

We designed (Fig. 1A) a suite of classification models relying on hidden representations encoded by protein language models (PLMs). Sequences are tokenized at the single amino acid level and embedded through multiple transformer layers, generating highly dimensional vector representations for each residue.

Exploiting the relatively large training datasets ($∼10^{5}-10^{6}$ distinct tokens for intrinsic disorder, $∼10^{7}$ for soft disorder), we implemented low-rank adaptation (LoRA) of the model, by introducing additional trainable layers. These layers learn low-rank decomposition of large weight matrix updates (Yu *et al.* 2023), enabling effective modification of the model internal representations. This approach is analogous to fine-tuning but involves training only a small fraction of the model parameters. By freezing pretrained parameters, LoRA prevents catastrophic forgetting while retaining the ability to disable adapter layers and revert to the original model. For all models, LoRA layers were added to the linear transformations in every multihead attention module of the architecture. Details on hyperparameter choices, specific to the pretrained model and classification task, are provided in the Materials and methods section.

In this scope, the detection of disordered residues is formulated as a binary token classification task. Using pretrained transformer architectures, we add a linear classification head with dropout, applied in parallel to the representation of each residue. As a result, LoRA-DR-suite  generates a profile of intrinsic/soft disorder for the input sequence, assigning each residue a probability score indicating its likelihood of being disordered (Fig. 1A, right).

In this study, we employed well-established PLMs as pretrained models, including two versions of the Evolutionary Scale Model (ESM2) with 35M and 650M parameters (Lin *et al.* 2023), ProtT5 from the ProtTrans suite (Elnaggar *et al.* 2022), and Ankh (Elnaggar *et al.* 2023). All models were trained using cross-entropy loss with balanced class weights to address class imbalance in the intrinsic and soft disorder datasets. These four models are integrated into the LoRA-DR-suite package.

### 2.2 Comparative performance analysis of LoRA-DR-suite on intrinsic disorder

The performance of LoRA-DR-suite models was evaluated against several established methods for intrinsic disorder prediction across three benchmark datasets: CAID1–DisProt (Necci *et al.* 2021), CAID2_NOX (Del Conte *et al.* 2023a, 2023b), and CAID3_NOX (https://caid.idpcentral.org/challenge). These datasets were introduced by the Critical Assessment of Intrinsic protein Disorder (CAID) challenge, a biannual competition to benchmark the state-of-the-art in predicting IDRs in proteins: CAID1-DisProt contains the NOX subset of the first CAID round consisting of 646 protein sequences, CAID2_NOX contains 210 protein sequences from CAID2, and CAID3_NOX comprises 148 protein sequences from CAID3. Protein sequences in the NOX datasets are labeled based on information derived from circular dichroism experiments (Fig. 1B). Key performance metrics include area under the receiver operating characteristic curve (ROC AUC), F1 score, Matthews correlation coefficient (MCC), and area under the precision recall curve (PR AUC) (see Materials and methods section), allowing for a comprehensive comparison of model efficacy. For each dataset, we compare with the best performing tools that participated to the corresponding CAID round. For the CAID1 and CAID2 evaluation, our models were trained exclusively on data from DisProt 7.0 database (models denoted with the suffix “DisProt7”). For the CAID3 evaluation, we retrained our models by expanding the training set with data from both CAID1 and CAID2 (models labeled with the suffix “ID”). Further details on training data and methodology are provided in the Materials and methods section. The performance of models trained on DisProt 7.0 database in the CAID3 evaluation is summarized in Supplementary Table S1.

On CAID1–DisProt test set (Table 1), ESM2_650M-LoRA achieved the best overall performance (ROC AUC 0.833, PR AUC 0.510), indicating strong discriminative power and precision–recall tradeoff. ESM2_35M-LoRA and Ankh-LoRA closely followed (ROC AUC 0.812 and 0.819), with marginal differences in F1 and MCC scores, underscoring the robustness of LoRA-enhanced models. These models notably outperformed approaches such as SPOT-Disorder2 (Hanson *et al.* 2019) and RawMSA (Mirabello and Wallner 2019), which lagged in all metrics. This highlights the advantage of using PLMs with low-rank adaptation for intrinsic disorder prediction.

**Table 1.** Results of fine-tuned models on CAID1-DisProt and CAID2_NOX test sets, compared with best performing tools for each challenge, based on ROC AUC metric.

Model	ROC AUC	F1	MCC	PR AUC
**CAID1-DisProt**				
ESM2_650M-LoRA-DisProt7	**0.833**	**0.498**	**0.394**	**0.510**
ESM2_35M-LoRA-DisProt7	0.812	0.463	0.350	0.505
Ankh-LoRA-DisProt7	0.819	0.490	0.380	0.499
ProtT5-LoRA-DisProt7	0.817	0.482	0.373	0.463
flDPnn	0.814	0.483	0.370	0.475
flDPlr	0.793	0.452	0.330	0.421
rawMSA	0.780	0.445	0.328	0.413
SPOT-Disorder2	0.760	0.470	0.349	0.340
** CAID2_NOX**				
ESM2_650M-LoRA-DisProt7	0.837	0.535	0.407	0.594
ESM2_35M-LoRA-DisProt7	0.813	0.534	0.406	0.571
Ankh-LoRA-DisProt7	0.838	**0.548**	**0.427**	0.556
ProtT5-LoRA-DisProt7	0.837	0.544	0.420	0.577
flDPnn2	0.838	0.526	0.414	**0.596**
flDPnn	0.835	0.521	0.409	0.581
Dispredict3	**0.842**	0.517	0.404	0.579
DisoPred	0.824	0.484	0.364	0.503
SPOT-Disorder2	0.760	0.506	0.303	0.544

See https://caid.idpcentral.org/challenge/results.

The CAID2_NOX results (Table 1) further demonstrate the competitiveness of LoRA-enhanced PLMs. Ankh-LoRA, ProtT5-LoRA, and ESM2_650M-LoRA achieved comparable ROC AUC scores (0.837–0.838), with Ankh-LoRA slightly leading in F1 score (0.548) and MCC (0.427). flDPnn2 (Wang *et al.* 2024) and Dispredict3 (Kabir and Hoque 2024) performed well in ROC AUC and PR AUC, remaining competitive alternatives. Again, LoRA-enabled transformers show effectiveness in balancing precision and recall across diverse datasets.

To ensure a fair comparison on the CAID3_NOX benchmark (Table 2), we filtered models based on the fraction of residues (positive or negative) they scored during the competition, as some lacked predictions for all proteins. We then reported metrics on CAID3_NOX dataset for the 5 best performing models with full residue coverage, and on the common subset of proteins scored by the top 10 models (115 proteins out of 148) corresponding to the 53.8% residue coverage. It is worth noticing that sequences in the restricted test set are shorter (median length 314 residues) than those removed (median length 1053 residues; Supplementary Table S2). Both experiments reveal the strong performance of LoRA-enhanced models, which predict intrinsic disorder across entire protein sequences. Among them, notably ESM2_650M-LoRA achieved top scores in ROC AUC (0.880), F1 score (0.649), MCC (0.524), and PR AUC (0.721). ESM2_35M-LoRA also performed competitively, highlighting scalability across transformer sizes. An analogous analysis was performed for the other CAID3 subsets—CAID3_PDB, CAID3_Binding, and CAID3_Linker—reported in Supplementary Tables S3–S5 respectively, where some proteins have been removed (Supplementary Table S6). While task-specific methods outperform LoRA models on CAID3_PDB and CAID3_Linker, LoRA-enhanced models achieve superior performance in binding prediction, surpassing dedicated predictors particularly in PR AUC (0.72 for ProtT5-LORA-ID) and Max F1 score (0.618 for esm2_650M-LORA-ID). Notably, for all LoRA models except Ankh-LoRA-ID, the threshold maximizing F1 in binding prediction exceeds 0.85, significantly higher than the 0.50–0.62 range observed for generic disorder prediction. This suggests that LoRA models can reliably identify putative binding regions without requiring task-specific training.

**Table 2.** Results of fine-tuned models on the CAID3_NOX test set.

Model	ROC	F1	MCC	PR	Max
	AUC			AUC	F1
**CAID3_NOX (100% residue coverage)**					
ESM2_650M-LoRA-ID	**0.880**	**0.649**	**0.523**	**0.721**	0.656
ESM2_35M-LoRA-ID	0.868	0.645	0.518	0.689	**0.659**
Ankh-LoRA-ID	0.861	0.637	0.507	0.684	0.638
ProtT5-LoRA-ID	0.862	0.628	0.494	0.698	0.633
rawMSA-disorder	0.858	0.504	0.399	0.640	0.640
flDPnn3a	0.851	0.587	0.436	0.687	0.642
rawMSA	0.847	0.606	0.462	0.656	0.614
UdonPred-combined	0.841	0.620	0.478	0.634	0.626
flDPnn3b	0.836	0.602	0.456	0.645	0.615
**CAID3_NOX (53.8% residue coverage)**					
ESM2_650M-LoRA-ID	**0.896**	**0.745**	**0.606**	**0.800**	**0.760**
ESM2_35M-LoRA-ID	0.880	0.730	0.583	0.765	0.752
Ankh-LoRA-ID	0.866	0.717	0.559	0.751	0.718
ProtT5-LoRA-ID	0.884	0.731	0.581	0.789	0.732
DisorderUnetLM	0.881	0.704	0.561	0.778	0.713
flDPnn3a	0.871	0.676	0.499	0.767	0.720
ESMDisPred-2PDB	0.864	0.572	0.459	0.740	0.728
DisoFLAG-IDR	0.863	0.690	0.538	0.761	0.701
ESMDisPred-2	0.857	0.605	0.477	0.738	0.718

F1 and MCC scores were calculated for comparative tools as they were not provided on the official results page https://caid.idpcentral.org/challenge/results. Performance is reported separately for models covering 100% of residues and for a reduced test set comprising 53.8% of residues, see main text. See https://caid.idpcentral.org/challenge/results.

Across all benchmarks, ESM2_650M-LoRA consistently demonstrated strong performance, particularly on CAID3_NOX, where additional data from CAID1 and CAID2 were used for training (Materials and methods section). Adding LoRA layers significantly enhanced the capacity of pretrained language models to predict intrinsically disordered residues with precision and robustness, even with class imbalance. Models like SPOT-Disorder2 and RawMSA lagged behind, whereas hybrid models such as flDPnn showed competitive but narrower advantages.

### 2.3 Extending the definition of disorder through the database SoftDis for soft disorder

The concept of soft disorder was introduced in (Seoane and Carbone 2021) as a general term for regions in a protein identified as flexible (characterized by a high B-factor) or intermittently missing across different X-ray crystal structures of the same sequence. Formally, this definition is derived from an extensive analysis of clusters of alternative structures for the same protein sequence in the Protein Data Bank (PDB): based on the same procedure introduced in (Seoane and Carbone 2021), we newly analyzed 229 370 crystallographic structures and clustered their 484 044 chains into 64 285 clusters to create the SoftDis database. A residue is classified as soft-disordered if it is flexible or missing in at least one crystal structure within a cluster, excluding residues that are consistently missing across all structures. Based on this definition, we statistically demonstrated a significant correlation between SDRs and interface sites observed in protein complexes of identical sequences (Seoane and Carbone 2021). This analysis also revealed that disordered regions often undergo structural shifts upon binding, facilitating the formation of new interfaces that arise during the assembly pathway (Seoane and Carbone 2022). Since all chains were retained during database construction, SoftDis encompasses short segments previously identified as MoRFs (Song and Kurgan 2025) and LIPs (Peng *et al.* 2023). While these segments are typically labeled as soft disorder, the soft disorder category covers a broader range. Supplementary Figures S1 and S2 illustrate SDR lengths (from 1 to >300 residues) and show that the number of SDRs per PDB chain can reach several dozen. The SoftDis database is available on the HuggingFace datasets Hub (see Data and Software Availability). It includes labels for representative sequences in clusters, covering soft disorder, missing residues, and interfaces as identified in the PDB. Reduced datasets based on similarity clustering and nonredundant splits designed for learning tasks are also included along with detailed data for chains within each cluster.

### 2.4 Soft disorder classification

Results for intrinsic disorder classification demonstrate PLMs fine-tuning effectively identifies disorder markers, prompting us to extend these models to the broader definition of soft disorder. In this scope, we evaluated the same suite of models on our newly constructed soft disorder dataset (details in the Materials and methods section). For learning and evaluation, we first reduced redundancy of training data by clustering representative sequences for each PDB cluster at 50% sequence identity, then randomly splitting the remaining sequences into train, validation and test sets. Sequences in validation and test sets were further filtered at a 30% sequence identity threshold to ensure generalizability. For each fine-tuned PLM of LoRA-DR-suite, the hyperparameters search was performed according to the same procedure as in the case of intrinsic disorder, but using a smaller subset (15%) of all training data for feasibility. Models with selected parameters were then re-trained on the complete dataset.

Table 3 provides a performance comparison of LoRA-DR-suite models for predicting soft disordered residues. The results reveal a tradeoff between model complexity and predictive performance: ESM2_650M-LoRA, with its larger size and higher computational demand, achieves the highest accuracy, whereas the smaller ESM2_35M-LoRA model remains computationally efficient with slightly reduced performance. Ankh-LoRA offers a balanced intermediate option. These results align with intrinsic disorder classification results, underscoring the predictive power of adapted embeddings in recognizing disorder patterns. Notably ESM-derived models excel despite their lower architectural complexity.

**Table 3.** Results of LoRA-DR-suite models trained on soft disorder on the SoftDis test set.

Model	ROC AUC	F1	MCC	PR AUC
ESM2_650M-LoRA-SD	**0.839**	**0.655**	**0.476**	**0.729**
ESM2_35M-LoRA-SD	0.812	0.626	0.430	0.690
Ankh-LoRA-SD	0.831	0.646	0.468	0.716
ProtT5-LoRA-SD	0.823	0.638	0.451	0.702

**Figure 2.**
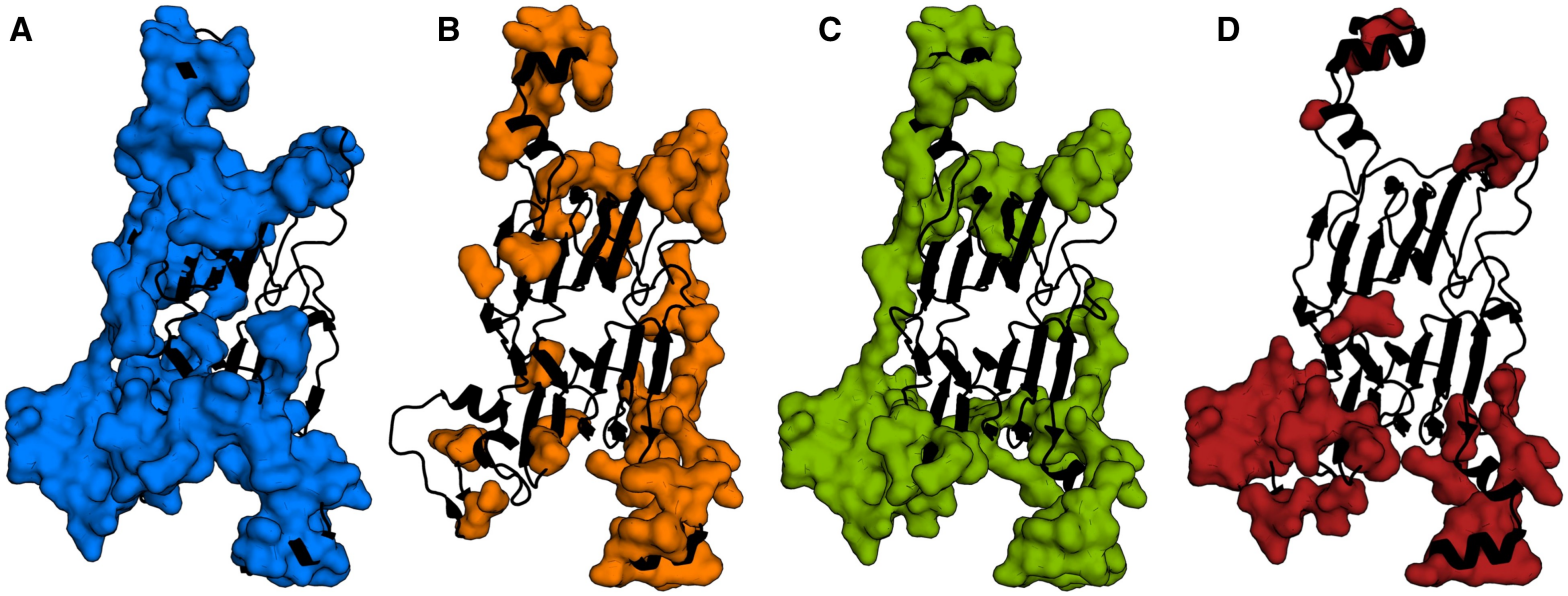
Structural analysis of the *Bacillus subtilis* Oxalate Decarboxylase OxdC protein. (A) Residues involved in binding sites across any complex in the SoftDis cluster containing PDB ID 2UYA are shown as surface (dark blue). (B) Soft disordered residues identified in all PDB complexes, as in (A), are shown as surface (orange). (C) Residues predicted as soft disordered by ESM2_650M-LoRA are shown as surface (green). (D) Residues with pLDDT scores below the chain’s average are shown as surface (red).

To illustrate the performance of LoRA-DR-suite on soft disorder predictions, we analyzed the *Bacillus subtilis* Oxalate Decarboxylase OxdC protein (PDB ID 2UYA). Experimental data from the SoftDis database were used to compare the union of interface residues (Fig. 2A) and the union of soft disordered residues (Fig. 2B) computed on all PDB complexes containing this chain to the predicted soft disorder residues (Fig. 2C). Notably, the predicted soft disordered residues show a high correlation with the experimental data. Additionally, low pLDDT values from the AlphaFold structural model of the protein (Fig. 2D) align with the predicted soft disordered residues (Jumper *et al.* 2021), further supporting the robustness of the LoRA-DR-suite’s predictions.

### 2.5 Linking soft and intrinsic disorder with pLDDT

**Figure 3.**
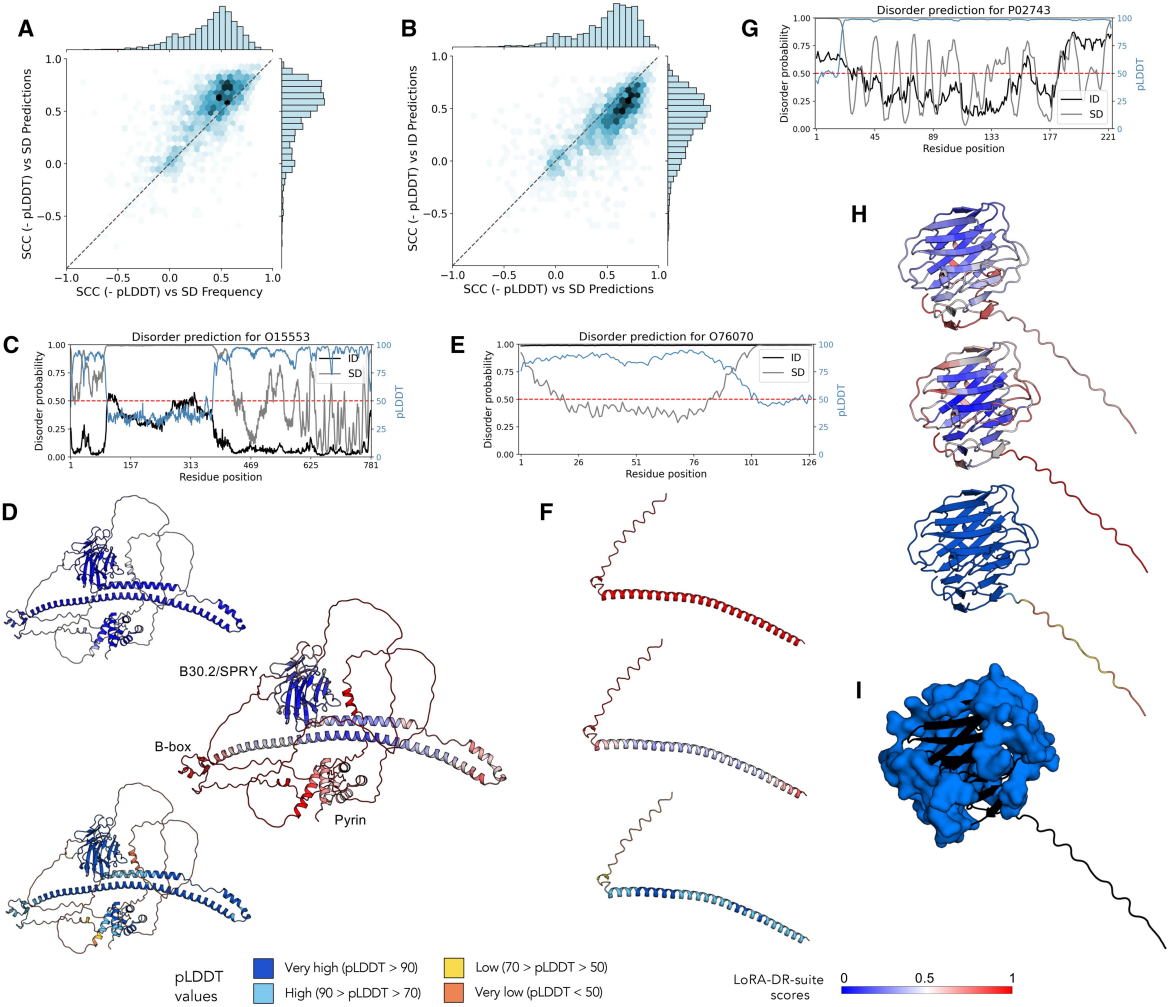
Structural analysis of the MEFV, Synuclein and SAMP human proteins. (A) Heatmap of similarity (measured by the Spearman Correlation Coefficient) between pLDDT scores and ESM2_650M-LoRA scores for soft disorder predictions, and between pLDDT scores and the frequency of a residue being soft disordered within the PDB cluster in the SoftDis database. The correlations are calculated on the negative of the pLDDT scores. (B) As in (A), heatmap between pLDDT scores and ESM2_650M-LoRA scores of intrinsic disorder, and pLDDT and ESM2_650M-LoRA scores of soft disorder predictions. (C) Profiles of ESM2_650M-LoRA scores of soft disorder, ESM2_650M-LoRA scores of intrinsic disorder, and pLDDT for the MEFV protein (UniProt ID O15553). (D) MEFV structural model in AlphaFold Database colored with ESM2_650M-LoRA intrinsic disorder scores (top), ESM2_650M-LoRA soft disorder scores (middle), and pLDDT confidence scores (bottom). (E) Profiles of ESM2_650M-LoRA scores of soft disorder, ESM2_650M-LoRA scores of intrinsic disorder, and pLDDT for the Synuclein protein (UniProt ID O76070). (F) Synucleins structural model in AlphaFold Database colored with ESM2_650M-LoRA intrinsic disorder scores (top), ESM2_650M-LoRA soft disorder scores (middle), and pLDDT confidence scores (bottom). (G) Profiles of ESM2_650M-LoRA scores of soft disorder, ESM2_650M-LoRA scores of intrinsic disorder, and pLDDT for the Serum amyloid P-component protein (SAMP; UniProt ID P02743). (H) AlphaFold model structure of the SAMP protein with residues colored by predicted scores of intrinsic disorder (top), predicted scores of soft disorder (middle), and pLDDT values (bottom). Corresponding color bars are shown on the right. (I) PDB structure 1GYK, chain A, with residues highlighted in blue to indicate interface residues identified in SoftDis. These residues were computed using the cluster represented by chain C of PDB structure 1SAC, which contains 90 chains.

We conducted a large-scale analysis on the 2009 chains from our SoftDis testing dataset that have structural models available in the AlphaFold database. This analysis reveals that lower pLDDT values—indicating lower structural confidence or higher disorder—correlate positively with soft disorder predictions and the frequency of soft disorder observed in similar protein chains (Fig. 3A). These results suggest that regions with low pLDDT scores align well with both predicted and observed soft disorder patterns, effectively capturing structural variability and flexibility. Figure 3B further shows that pLDDT scores align with LoRA-DR-suite predictions for both intrinsic and soft disorder. However, the correlation is stronger for soft disorder, highlighting key distinctions in how these two disorder types relate to structural confidence (Ruff and Pappu 2021).

Figure 3C and D focus on the Mediterranean Fever (MEFV) human protein gene, an important modulator of innate immunity. The soft disorder and pLDDT profiles exhibit a strong Spearman correlation (0.865 with -pLDDT), with soft disorder effectively capturing flexibility differences across annotated domains that are not detected as intrinsic disorder. For example, the pyrin domain (1–92) and B-box domain (370–412) are identified as highly flexible, whereas the B30.2/SPRY domain (580–775) appears much more stable. Additionally, the long helices (420–582), required for homotrimerization and pyroptosome induction, exhibit variable flexibility: stable at the center (low soft disorder, high pLDDT) but highly flexible at the helix extremes (high soft disorder, low pLDDT). These patterns are undetected by intrinsic disorder predictions, which correlate weakly with -pLDDT (0.796).

Figure 3E and F analyze the Synuclein protein. While our intrinsic disorder predictor classifies it as highly disordered, the soft disorder profile reveals a more detailed perspective: the helix’s extremes are identified as highly flexible, while the central region is stable. -pLDDT strongly aligns with soft disorder (correlation of 0.816), but weakly with intrinsic disorder (0.505), underscoring differences between disorder types.

Figure 3G and H analyze the Serum amyloid P-component protein. This protein exhibits consistently high pLDDT values across its sequence, with correlations of 0.591 and 0.829 observed between -pLDDT and intrinsic disorder and soft disorder, respectively. The soft disorder profile identifies several regions with high scores, indicative of flexible zones. These regions are localized on the protein surface (Fig. 3H, center) and correspond to interaction sites, as highlighted by the blue residues in Fig. 3I, derived from the SoftDis database.

These observations underscore the relevance of soft disorder predictions in identifying structurally flexible, functionally important regions, involved in protein–protein interactions. The strong correlation with -pLDDT further highlights the potential of combining structural confidence scores with soft disorder profiles to enhance our understanding of protein flexibility, structural variability, and interaction dynamics.

### 2.6 Adapted embeddings better align with disorder

LoRA layers alter the internal representations and output embeddings of the models. To evaluate how these changes affect disorder classification, we trained logistic regression classifiers on embeddings from both the pretrained and LoRA-adapted ESM2_650M models. For intrinsic disorder, we used the CAID3_NOX dataset; for soft disorder, we selected a random subset of 500 proteins from the SoftDis test set to avoid biases from the LoRA training data. Each dataset was split: 70% of sequences were used for five-fold cross-validation (optimized via ROC AUC across varying regularization strengths), and 30% were reserved for independent testing. As shown in Supplementary Table S7, LoRA-adapted embeddings substantially improve classification performance on both tasks, with an ROC AUC of 0.881 and 0.817 for intrinsic and soft disorder respectively, versus 0.832 and 0.784 for ESM2_650M.

### 2.7 Adapted embeddings influence on attention scores

LoRA-DR-suite models exploit adapted embeddings to extract and distinguish fundamental differences between disorder signals. To explore whether intrinsic and soft disorder residues are primarily influenced by global or local effects learned via adapter layers, we used contact prediction as an indirect approach to analyze changes in attention scores after model fine-tuning (Rao *et al.* 2020).

**Figure 4.**
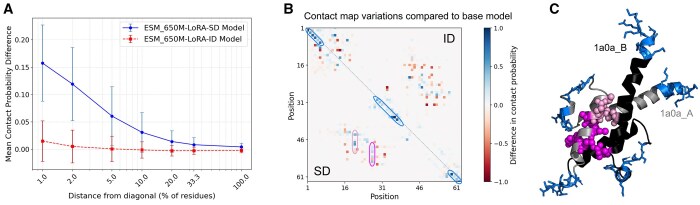
Variations in predicted contacts maps induced by LoRA layers. (A) Average variation in contacts probability scores returned by ESM2_650M contact prediction head, between models fine-tuned on soft disorder (ESM2_650M-LoRA-SD) and intrinsic disorder (ESM2_650M-LoRA-ID) with respect to the ESM2_650M base model, at different distances from the principal diagonal (measured as fraction of total protein length). (B) Example of contact map variations induced by fine-tuned models on soft disorder (SD, below diagonal) and intrinsic disorder (ID, above diagonal) for PDB chain 1A0A_B. (C) Enhanced predicted contacts from soft disorder fine-tuning are plotted on the dimeric PDB structure (1A0A; DNA chains are removed). Positions with increased scores near the diagonal are colored in blue on both chains. Residues showing enhanced contacts with positions 20 and 27 from chain 1A0A_B are colored in pink and magenta, respectively, in chain 1A0A_A.

We performed a large-scale analysis of variations in predicted contact maps across 2512 chains from the SoftDis test set, excluding those longer than 1024 residues, to assess the impact of LoRA layers compared to the base model ESM2_650M. Adapted embeddings were generated by training with information from either intrinsic or soft disorder, while contact probabilities were derived from the pretrained ESM2_650M contact prediction head on attention outputs. Figure 4A shows that ESM2_650M-LoRA for intrinsic disorder induces variations primarily at very short ranges. In contrast, training on soft disorder leads to variations observed at much greater distances, extending up to a third of the protein’s length. Although the number of long-range variations decreases with increasing distance, they remain consistently present. Figure 4B presents an in-depth analysis of the phosphate system positive regulator protein PHO4 (chain B), whose dimer is known to form a complex with DNA. The variability in contact probabilities is significantly more pronounced when training on soft disorder, with notable differences observable (see blue squares) at short range compared to embeddings trained on intrinsic disorder. For intrinsic disorder, adapted embeddings tend to loose contact information at larger ranges (see red squares), a phenomenon also present for soft disorder but to a lesser extent. In Fig. 4C, positions with enhanced contact scores for the model trained on soft disorder are shown. High scores near the principal diagonal (blue) are mostly found in coiled regions, while those at larger distances reveal contacts with a partner chain in the dimeric complex. Notably, residues predicted to contact alanine at position 20 (pink) and leucine at position 27 (magenta) are highlighted. These results emphasize the unique influence of soft disorder training on capturing contact probabilities through adapted embeddings.

## 3 Materials and methods

### 3.1 Intrinsic disorder datasets

For model selection and training, we used the dataset from Hu *et al.* (2021), derived from DisProt 7.0 (Piovesan *et al.* 2017, Hatos *et al.* 2020, Aspromonte *et al.* 2024) following identical preprocessing. The original dataset comprised 745 experimentally labeled sequences, split into training (445 sequences), validation (100 sequences), and test (200 sequences) sets. To ensure low similarity between training and test sequences, the combined training and test sets were clustered at 25% sequence identity using the CD-HIT algorithm (Huang *et al.* 2010). Test sequences belonging to the same clusters as training sequences were excluded, leaving a final test set of 176 low-similarity sequences.

To evaluate the generalization capabilities of the models, we also used datasets from the first, second, and third editions of the CAID challenge. The CAID1-DisProt dataset contains 646 protein sequences that were unknown to the contestants before the evaluation, and whose labels follow the DisProt format. Proteins were sourced from diverse species, including humans and other mammals (368 sequences) as well as prokaryotes (77 sequences). Similarly, the CAID2_NOX dataset consists of 210 sequences from the Disorder_NOX subset of the second challenge edition, while the CAID3_NOX includes 148 sequences from the Disorder_NOX subset of the third edition. Moreover, in the CAID3 evaluation, we added all sequences from CAID1-DisProt and CAID2_NOX, as well as the initially held out set, to the training data, while keeping the original 100 sequences for validation. Sequences in the training and validation sets were truncated to a maximum length of 1024 residues in all the experiments. See the legend of Fig. 1B for the definition of intrinsic disorder, CAID_NOX and CAID_PDB.

### 3.2 Construction of the soft disorder database SoftDis

To construct the SoftDis database, we retrieved protein structures from the PDB archive (snapshot as of 15 October 2024) and clustered sequences using MMSeqs2 (Steinegger and Söding 2017, Kallenborn *et al.* 2024) at 90% sequence identity and 90% coverage. This process yielded 64 285 clusters encompassing a total of 484 044 chains belonging to 229 376 structures. The average number of structures per cluster was 7.53, with a median of 3. Nearly half of the clusters (31 412) contained only 1 or 2 sequences, while the largest cluster included 1727 homologs.

The representative chain for each cluster was selected from the experimental structure with the highest resolution or best R-value. For protein complexes, individual chains were analyzed separately and assigned to their respective clusters.

Residues in each chain were labeled as missing if annotated under REMARK 465 in the PDB file. A residue was classified as soft disordered if it was either missing or had a normalized B-factor $b_{i}=(B_{i}-\bar{B})/\sigma_{B}>3$, where $B_{i}$ represents the B-factor of the $C_{\alpha }$ atom, $\bar{B}$ and $\sigma_{B}$ are the mean and standard deviation of $B_{i}$ values within the chain. Additionally, residues were labeled as interface if they participated in protein–protein or protein–DNA/RNA interactions. Protein–protein binding sites were identified using the INTerface Builder tool (Dequeker *et al.* 2017), where residue contacts are defined by $C_{\alpha }$ atoms within 5 Å. Protein–DNA/RNA binding sites were determined as residues showing decreased accessible surface area, measured using Naccess (Hubbard and Thornton 1993) with a 1.4 Å  probe, upon binding.

For each site in the representative sequence of a cluster, we recorded how often it was labeled as soft disordered across all chains in the cluster, excluding consistently missing sites. Similarly, we noted how often each site was labeled as an interface. Figure 1C illustrates this labeling process for soft disordered residues, missing residues, intrinsically disordered residues, and disorder-to-order transition residues in the SoftDis database.

To adapt the dataset for machine learning pipelines, sequences shorter than 20 or longer than 2048 amino acids were removed, and redundancy was reduced by clustering sequences at 50% identity and 80% coverage. The resulting 38 218 sequences were randomly split into training (70%), validation (10%), and test (20%) sets. Validation and test sets were then pruned at different sequence identity thresholds (0.9, 0.7, 0.5, and 0.3) with respect to the training set, enabling progressively challenging evaluations. For training and evaluation, we used the dataset pruned at the strictest (0.3) threshold, resulting in 26 752 training, 1155 validation, and 2523 test sequences.

To generate binary soft disorder labels, residues are assigned 1 if marked as soft disordered in at least one sequence in the cluster, and 0 otherwise. On average, 32% of residues per sequence are labeled as soft disordered across splits. We also provide the fraction of chains where each residue is identified as soft disordered, enabling model uncertainty quantification. A similar analysis applied to residues at protein–protein or protein–DNA/RNA interaction sites supports multilabel classification at each position.

### 3.3 Evaluation metrics

To properly evaluate LoRA-DR-suite performance, we rely on a ground truth set by reference datasets, depending on the experiment, and the following quantities: known disordered residues identified by LoRA-DR-suite (true positives, TP), disordered residues identified by LoRA-DR-suite which are not known (false positives, FP), disordered residues that are not found by LoRA-DR-suite but are known (false negatives, FN), and disordered residues that are not known and are not detected by LoRA-DR-suite (true negatives, TN).

Due to label imbalance, we considered the following metrics:

- ROC AUC shows the tradeoff between true positive rate and false positive rate across different decision thresholds.
- Matthews correlation coefficient: MCC =(TP·TN−FP·FN)/K, where $K=\sqrt{\left(TP+FP)(TP+FN)(TN+FP)(TN+FN\right)}$
- F1 score =2TP/(2TP+FP+FN)
- PR AUC, computed as average precision, where the precision–recall metric shows the tradeoff between precision and recall for different thresholds, with recall (sensitivity) =TP/(TP+FN) and precision (positive predictive value) =TP/(TP+FP).
- Max F1 score, corresponding to the maximum F1 score computed at different thresholds with a step of 0.01.

### 3.4 PLMs fine-tuning and hyperparameters selection

Pretrained PLM checkpoints used in our evaluation were taken from the HuggingFace Hub, enabling easy customization for parameter-efficient fine-tuning strategies such as LoRA on attention layers. Hyperparameter optimization was performed using the HuggingFace Trainer and Optuna backend (Akiba *et al.* 2019), with the following possible choices across all experiments:

- discrete parameters:
  - LoRA rank r∈{8,16,32};
  - dropout probability p∈{0.1,0.2,0.3};
  - scheduler warm up ratio ∈{0.1,0.2};
  - optimizer weight decay ∈{0.1,0.01,0.001}.
- Maximum learning rate $\text{lr}\in [10^{-5},10^{-3}]$, with uniform probability on logarithmic scale.
- Attention layers with LoRA adapters, {Q, K, V} or {Q, V}.

Optimizer was set to AdamW (Loshchilov 2017) with default parameters and cosine scheduler decay. We used an effective batch size of 8 for training on Disprot 7.0, and of 16 for training on the full intrinsic disorder and soft disorder datasets.

For each experiment, the models with the highest ROC AUC on the validation set were selected from 40 trials using random parameter search (Supplementary Table S8). Models were trained for up to 10 epochs, with early stopping regularization based on validation ROC AUC and patience set to 2. Training and evaluations were performed on a single computing node equipped with an Intel Xeon Gold 6330 processor (4 cores, 64 GB RAM reserved) and a NVIDIA A100 PCIe GPU with 80GB RAM (see Supplementary Table S9 for evaluation times on CAID datasets).

## 4 Discussion

By incorporating LoRA adaptations into PLMs, LoRA-DR-suite achieves performance that matches or surpasses state-of-the-art methods, positioning itself as a highly competitive tool for intrinsic disorder prediction. Its versatility allows it to effectively address both intrinsic and soft disorder, broadening its applicability and paving the way for new research directions in the field.

*Why a predictor of soft disorder?* By predicting regions of flexibility or transient disorder across ensembles of related proteins, LoRA-DR-suite models provide valuable insights in the following areas:

1. Identification of protein interactions sites. Protein interfaces are critical regions, often sensitive to mutations and linked to pathogenic variants. IDRs enable diverse binding modes, facilitating regulation, recognition, and signaling through high-specificity, low-affinity interactions (Uversky 2018). SDRs further enrich interface complexity, with residues of defined interface roles—transient or permanent—that can dynamically shift between structured and disordered states (Seoane and Carbone 2022). Hence, differentiating intrinsic disorder, soft disorder, and other residues is key to understanding protein interfaces, and the integration of these concepts into models of protein–protein interactions and protein dynamics holds the potential for significant breakthroughs.
2. Link between disorder and assembly pathways. Soft disorder reveals how structural flexibility shapes protein interactions and functional networks (Seoane and Carbone 2022). Thus, soft disorder-informed predictions can serve as a foundation for designing network-targeted therapeutics exploiting the unique properties of SDRs. In addition, the connection between soft disorder and assembly may reveal how proteins exploit flexibility and transient disorder in multivalent interactions.
3. Guidance for experimental studies. Accurate and fast sequence-based predictions help to efficiently screen large sequence datasets and prioritize candidate residues for validation, reducing reliance on costly and time-intensive methods for structural determination.

*Expanding protein disorder research through multiple disorders.* Integrating intrinsic disorder—defined via circular dichroism on isolated protein chains—with soft disorder—identified through flexibility and missing residues across similar X-ray structures—broadens disorder research in many ways.

Indeed, identifying factors underlying the overlap and divergence between the two notions of disorder, as predicted by LoRA-DR-suite and annotated in SoftDis, could reveal their distinct functional roles in signaling, regulation, and scaffolding, and uncover the interplay between structural and environmental factors that shape disorder dynamics. Furthermore, exploring the evolutionary constraints shaping disorder types across species or protein families could highlight distinct selective pressures driving protein functional diversity (Peng *et al.* 2015).

Future research in these directions is critical to understanding how intrinsic and soft disorder contribute to human diseases, where disruptions in disorder-mediated interactions drive pathological states (Uversky 2018). Studying intrinsically disordered and hybrid proteins—comprising ordered domains and functional IDRs exhibiting missing, disordered-to-ordered, consistently flexible, or flexible-to-rigid residues—may yield insights into disease mechanisms and novel therapeutic targets.

*Repurposable adapted embeddings*. Although not explored here, the adapted embeddings produced by the models can be extracted, analyzed, or repurposed for other tasks related to intrinsic or soft disorder in proteins. The prediction of conditions or partners that induce disorder-to-order transitions in SDRs is a promising research direction that would likely benefit from LoRA-DR-suite models, connecting predictive modeling with functional insights.

## Supplementary Material

btaf185_Supplementary_Data

## Data Availability

LoRA-DR-suite models and SoftDis dataset are available at https://huggingface.co/CQSB, with sample scripts for model deployment, dataset loading, and processing. Scripts for hyperparameters optimization and models testing are available at http://gitlab.lcqb.upmc.fr/lombardi/LoRA-DR-suite.
