# Supplementary material for: LoRA-DR-suite: adapted embeddings predict intrinsic and soft disorder from protein sequences
Source: Bioinformatics. 2025 Jul 15;41(Suppl 1):i439–48. doi: 10.1093/bioinformatics/btaf185 (PMC12261480; doi:10.1093/bioinformatics/btaf185)
Supplement: btaf185_Supplementary_Data [file btaf185_supplementary_data.zip › Carbone.211.sup.1.pdf]

# Supplementary Tables and Figures

| Model | ROC AUC | F1 | MCC | PR AUC | Max F1 |
|---|---|---|---|---|---|
| esm2_650M-LoRA-DisProt7 | 0.847 | 0.587 | 0.433 | 0.675 | 0.610 |
| esm2_35M-LoRA-DisProt7 | 0.856 | 0.629 | 0.497 | 0.658 | 0.647 |
| Ankh-LoRA-DisProt7 | 0.827 | 0.590 | 0.438 | 0.634 | 0.590 |
| ProtT5-LoRA-DisProt7 | 0.823 | 0.580 | 0.422 | 0.582 | 0.584 |

**Table S1.** Results of LoRA-DR-suite models trained on DisProt 7.0 dataset on CAID3_NOX test set.

| DisProt ID | Length (in aa) | Fraction of positive labels |
|---|---|---|
| DP03745 | 145 | 0.241 |
| DP03802 | 2905 | 0.149 |
| DP03803 | 1043 | 0.015 |
| DP03818 | 13 | 1.000 |
| DP03819 | 13 | 1.000 |
| DP03824 | 1136 | 0.242 |
| DP03826 | 1138 | 0.182 |
| DP03927 | 1084 | 0.056 |
| DP03930 | 852 | 0.147 |
| DP03931 | 854 | 0.063 |
| DP04005 | 3088 | 0.007 |
| DP04126 | 736 | 0.016 |
| DP04128 | 296 | 0.145 |
| DP04130 | 108 | 0.231 |
| DP04132 | 230 | 0.078 |
| DP04133 | 317 | 0.262 |
| DP04134 | 140 | 0.807 |
| DP04139 | 2285 | 0.658 |
| DP04155 | 1068 | 0.071 |
| DP04158 | 1292 | 0.013 |
| DP04162 | 1103 | 0.198 |
| DP04164 | 1247 | 0.136 |
| DP04166 | 955 | 0.015 |
| DP04178 | 1132 | 0.148 |
| DP04187 | 94 | 0.426 |
| DP04191 | 1162 | 0.057 |
| DP04197 | 1447 | 0.345 |
| DP04198 | 3010 | 0.013 |
| DP04217 | 1053 | 0.153 |
| DP04218 | 561 | 0.212 |
| DP04225 | 1799 | 0.028 |
| DP04232 | 1104 | 0.028 |
| DP04246 | 218 | 0.335 |

**Table S2.** Proteins from CAID3_NOX dataset that were not included in the reduced test set, as they were not evaluated by at least one of the top 10 models.

| Model | ROC AUC | F1 | MCC | PR AUC | Max F1 |
|---|---|---|---|---|---|
| CAID3_PDB (100% residue coverage) | | | | | |
| ESM2_650M-LoRA-ID | 0.929 | 0.780 | 0.695 | 0.882 | 0.783 |
| ESM2_35M-LoRA-ID | 0.918 | 0.775 | 0.680 | 0.859 | 0.777 |
| Ankh-LoRA-ID | 0.898 | 0.741 | 0.640 | 0.834 | 0.752 |
| ProtT5-LoRA-ID | 0.911 | 0.753 | 0.656 | 0.851 | 0.755 |
| AlphaFold3-rsa | **0.949** | **0.852** | **0.786** | **0.906** | **0.853** |
| LMDisorder | 0.941 | 0.812 | 0.724 | 0.897 | 0.824 |
| PredIDR2-Seq-Art | 0.939 | 0.789 | 0.689 | 0.885 | 0.801 |
| PredIDR2-Prof-Art | 0.935 | 0.777 | 0.671 | 0.868 | 0.785 |
| PredIDR2-Seq-Rnd | 0.933 | 0.768 | 0.658 | 0.869 | 0.788 |
| SETH-0 | 0.933 | 0.843 | 0.777 | 0.905 | 0.843 |
| CAID3_PDB (82.7% residue coverage) | | | | | |
| esm2_650M-LoRA-ID | 0.940 | 0.819 | 0.742 | 0.906 | 0.820 |
| esm2_35M-LoRA-ID | 0.921 | 0.798 | 0.718 | 0.872 | 0.799 |
| Ankh-LoRA-ID | 0.910 | 0.781 | 0.686 | 0.857 | 0.787 |
| ProtT5-LoRA-ID | 0.925 | 0.792 | 0.704 | 0.875 | 0.795 |
| PUNCH2 | **0.952** | **0.853** | **0.795** | **0.917** | **0.853** |
| PUNCH2-Light | 0.946 | 0.850 | 0.791 | 0.913 | 0.851 |
| SPOT-Disorder2 | 0.945 | 0.827 | 0.761 | 0.906 | 0.827 |
| PredIDR2-Prof-Art | 0.943 | 0.796 | 0.701 | 0.880 | 0.797 |
| AlphaFold-rsa | 0.942 | 0.835 | 0.762 | 0.896 | 0.839 |

**Table S3.** Results of LoRA-DR-suite models on the CAID3_PDB test set, on all dataset (considering only models with coverage higher than 95%) and on common proteins scored by top 10 models (206 out of 232 proteins, corresponding coverage 82.7%). See Table S6.

| Model | ROC AUC | F1 | MCC | PR AUC | Max F1 |
|---|---|---|---|---|---|
| CAID3_Binding (100% residue coverage) | | | | | |
| esm2_650M-LORA-ID | 0.891 | 0.503 | 0.424 | 0.706 | **0.618** |
| esm2_35M-LORA-ID | 0.885 | 0.511 | 0.441 | 0.622 | 0.617 |
| Ankh-LORA-ID | 0.875 | 0.525 | 0.442 | 0.614 | 0.577 |
| ProtT5-LORA-ID | **0.894** | **0.520** | **0.443** | **0.720** | 0.617 |
| IPA-All-bind | 0.856 | 0.500 | 0.424 | 0.552 | 0.589 |
| IPA-Nucleotide-bind | 0.850 | 0.522 | 0.429 | 0.536 | 0.530 |
| IPA-Protein-bind | 0.850 | 0.492 | 0.407 | 0.518 | 0.545 |
| AlphaFold3-binding | 0.828 | 0.464 | 0.384 | 0.399 | 0.506 |
| MoRFchibi-light | 0.802 | 0.342 | 0.289 | 0.422 | 0.477 |
| CAID3_Binding (81.2% residue coverage) | | | | | |
| esm2_650M-LoRA-ID | 0.885 | 0.498 | 0.399 | 0.729 | **0.644** |
| esm2_35M-LoRA-ID | 0.880 | 0.504 | 0.416 | 0.641 | 0.639 |
| Ankh-LoRA-ID | 0.864 | 0.517 | 0.415 | 0.632 | 0.584 |
| ProtT5-LoRA-ID | **0.893** | 0.518 | 0.424 | **0.753** | **0.644** |
| DisoFLAG-LB | 0.867 | 0.386 | **0.450** | 0.684 | 0.591 |
| DisoFLAG-IDR | 0.850 | **0.529** | 0.417 | 0.614 | 0.561 |
| IPA-Nucleotide-bind | 0.843 | 0.528 | 0.423 | 0.569 | 0.548 |
| IPA-All-bind | 0.841 | 0.491 | 0.395 | 0.562 | 0.598 |
| DisoFLAG-IB | 0.838 | 0.239 | 0.338 | 0.632 | 0.551 |

**Table S4.** Results of LoRA-DR-suite models on the CAID3_Binding test set. Top: on the all dataset (we considered models with coverage higher than 95%). Bottom: on the dataset comprising proteins scored by all the top 10 models (45 out of 51 proteins, corresponding coverage 81.2%). See Table S6 for the discarded proteins.

| Model | ROC AUC | F1 | MCC | PR AUC | Max F1 |
|---|---|---|---|---|---|
| CAID3_Linker (100% residue coverage) | | | | | |
| esm2_650M-LORA-ID | 0.852 | 0.370 | 0.366 | 0.258 | 0.390 |
| esm2_35M-LORA-ID | 0.838 | 0.378 | **0.376** | 0.233 | 0.430 |
| Ankh-LORA-ID | 0.849 | 0.398 | 0.387 | 0.240 | 0.408 |
| ProtT5-LORA-ID | 0.811 | 0.306 | 0.271 | 0.176 | 0.310 |
| LINKER-Pred2 | **0.854** | **0.392** | 0.352 | **0.359** | **0.485** |
| DisorderUnetLM | 0.851 | 0.369 | 0.338 | 0.258 | 0.374 |
| LINKER-Pred | 0.845 | 0.405 | 0.365 | 0.361 | 0.478 |
| flDPnn3b | 0.840 | 0.299 | 0.300 | 0.211 | 0.362 |
| flDPnn3a | 0.836 | 0.302 | 0.305 | 0.217 | 0.373 |
| CAID3_Linker (79.8% residue coverage) | | | | | |
| esm2_650M-LoRA-ID | 0.853 | 0.392 | 0.379 | 0.279 | 0.411 |
| esm2_35M-LoRA-ID | 0.834 | 0.388 | 0.379 | 0.242 | 0.436 |
| Ankh-LoRA-ID | 0.847 | 0.407 | 0.388 | 0.248 | 0.416 |
| ProtT5-LoRA-ID | 0.810 | 0.324 | 0.276 | 0.195 | 0.330 |
| IPA-AF2-Linker | **0.883** | **0.450** | 0.411 | 0.389 | 0.467 |
| LINKER-Pred2 | 0.882 | 0.426 | 0.378 | 0.413 | **0.517** |
| LINKER-Pred | 0.873 | 0.440 | 0.391 | **0.414** | 0.510 |
| DisoFLAG-IDR | 0.862 | 0.448 | **0.416** | 0.327 | 0.450 |
| LINKER-Pred-Lite | 0.853 | 0.433 | 0.383 | 0.421 | 0.459 |

**Table S5.** Results of LoRA-DR-suite models on the CAID3_Linker test set. Top: on the all dataset (we considered models with coverage higher than 95%). Bottom: on the dataset comprising proteins scored by all the top 10 models (19 out of 20 proteins, corresponding coverage 79.8%). See Table S6 for the discarded proteins.

| DisProt ID | Length (in aa) | Fraction of positive labels |
|---|---|---|
| | CAID3_PDB | |
| DP02732 | 1684 | 0.377 |
| DP03745 | 145 | 0.289 |
| DP03802 | 2905 | 0.371 |
| DP03803 | 1043 | 0.061 |
| DP03818 | 13 | 1.000 |
| DP03819 | 13 | 1.000 |
| DP03938 | 473 | 1.000 |
| DP03972 | 1735 | 0.029 |
| DP03980 | 912 | 1.000 |
| DP04005 | 3088 | 1.000 |
| DP04052 | 3056 | 0.010 |
| DP04057 | 1093 | 0.140 |
| DP04062 | 1507 | 0.012 |
| DP04064 | 814 | 1.000 |
| DP04096 | 1844 | 0.063 |
| DP04134 | 140 | 1.000 |
| DP04139 | 2285 | 0.726 |
| DP04163 | 409 | 1.000 |
| DP04184 | 146 | 0.275 |
| DP04190 | 88 | 1.000 |
| DP04197 | 1447 | 0.860 |
| DP04198 | 3010 | 0.032 |
| DP04208 | 274 | 0.184 |
| DP04225 | 1799 | 1.000 |
| DP04239 | 1430 | 0.093 |
| DP04241 | 467 | 1.000 |
| | CAID3_Binding | |
| DP03818 | 13 | 1.000 |
| DP03980 | 912 | 0.138 |
| DP04134 | 140 | 0.271 |
| DP04190 | 88 | 0.602 |
| DP04198 | 3010 | 0.013 |
| DP04241 | 467 | 0.385 |
| | CAID3_Linker | |
| DP04198 | 3010 | 0.013 |

**Table S6.** Proteins from CAID3_PDB, CAID3_Binding and CAID3_Linker datasets that were not included in the respective test sets, as they were not evaluated by at least one of the top 10 models.

| Model | ROC AUC | F1 | MCC | PR AUC |
|---|---|---|---|---|
| | | Intrinsic disorder | | |
| ESM2_650M-LoRA-ID | **0.881** | **0.625** | **0.506** | **0.748** |
| ESM2_650M | 0.832 | 0.565 | 0.416 | 0.632 |
| | | Soft disorder | | |
| ESM2_650M-LoRA-SD | **0.817** | **0.626** | **0.439** | **0.694** |
| ESM2_650M | 0.784 | 0.593 | 0.390 | 0.650 |

**Table S7.** Performances of logistic regression classifiers trained on model embeddings (either ESM alone or with LoRA) on held-out test sets.

| Model | LoRA $r$ | Dropout $p$ | LoRA layers | Learning rate | Warm up ratio | Weight decay | ROC AUC |
|---|---|---|---|---|---|---|---|
| ESM2_650M-LoRA-DisProt7 | 16 | 0.1 | $Q$, $V$ | $4.11 \cdot 10^{-4}$ | 0.1 | 0.1 | 0.842 |
| ESM2_35M-LoRA-DisProt7 | 32 | 0.3 | $Q$, $V$ | $7.09 \cdot 10^{-4}$ | 0.1 | 0.1 | 0.836 |
| Ankh-LoRA-DisProt7 | 16 | 0.3 | $Q$, $V$ | $5.64 \cdot 10^{-4}$ | 0.1 | 0.001 | 0.831 |
| ProtT5-LoRA-DisProt7 | 16 | 0.3 | $Q$, $V$ | $9.74 \cdot 10^{-4}$ | 0.1 | 0.01 | 0.853 |
| ESM2_650M-LoRA-ID | 32 | 0.1 | $Q$, $V$ | $2.08 \cdot 10^{-4}$ | 0.1 | 0.001 | 0.858 |
| ESM2_35M-LoRA-ID | 16 | 0.3 | $Q$, $K$, $V$ | $9.37 \cdot 10^{-4}$ | 0.2 | 0.001 | 0.848 |
| Ankh-LoRA-ID | 32 | 0.3 | $Q$, $V$ | $2.41 \cdot 10^{-4}$ | 0.2 | 0.01 | 0.836 |
| ProtT5-LoRA-ID | 8 | 0.2 | $Q$, $V$ | $7.73 \cdot 10^{-4}$ | 0.1 | 0.1 | 0.847 |
| ESM2_650M-LoRA-SD | 32 | 0.3 | $Q$, $V$ | $5.49 \cdot 10^{-4}$ | 0.2 | 0.01 | 0.844 |
| ESM2_35M-LoRA-SD | 16 | 0.1 | $Q$, $V$ | $5.80 \cdot 10^{-4}$ | 0.1 | 0.01 | 0.815 |
| Ankh-LoRA-SD | 8 | 0.1 | $Q$, $K$, $V$ | $8.09 \cdot 10^{-4}$ | 0.1 | 0.01 | 0.837 |
| ProtT5-LoRA-SD | 16 | 0.2 | $Q$, $V$ | $9.93 \cdot 10^{-4}$ | 0.1 | 0.001 | 0.826 |

**Table S8.** Hyperparameters selected for each model, and ROC AUC score obtained on the relative validation set. DisProt7 suffix refers to models evaluated on the first and second edition of CAID, thus trained only on the DisProt 7.0 subset, while models with ID (SD) suffix are trained on the full intrinsic disorder (soft disorder) datasets.

| Model | CAID1_Disprot | CAID2_NOX | CAID3_NOX |
|---|---|---|---|
| ESM2_650M-LoRA-ID | 0.161 (2.203) | 0.248 (4.132) | 0.154 (2.151) |
| ESM2_35M-LoRA-ID | 0.025 (0.360) | 0.041 (0.776) | 0.038 (0.358) |
| Ankh-LoRA-ID | 0.151 (3.436) | 0.222 (5.988) | 0.262 (3.367) |
| Ankh-ProtT5-ID | 0.272 (4.032) | 0.449 (7.143) | 0.262 (3.922) |
| Average $L$ | 522 | 766 | 492 |

**Table S9.** Average execution time per protein sequence (in seconds) for LoRA-DR-suite models on the evaluation sets for intrinsic disorder. Test samples were processed in single batches. For each dataset, we report the average length $L$ of the proteins. Times are reported for GPU (CPU) execution. See text for hardware specifics.

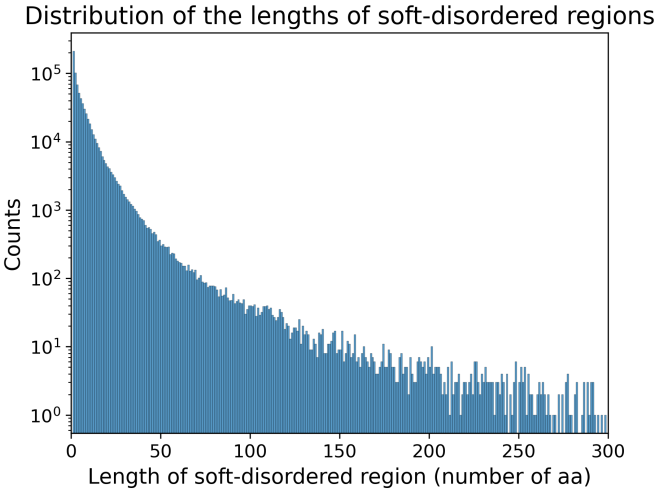


Fig. S1: Distribution of the length of soft-disordered regions in SoftDis clustered dataset.

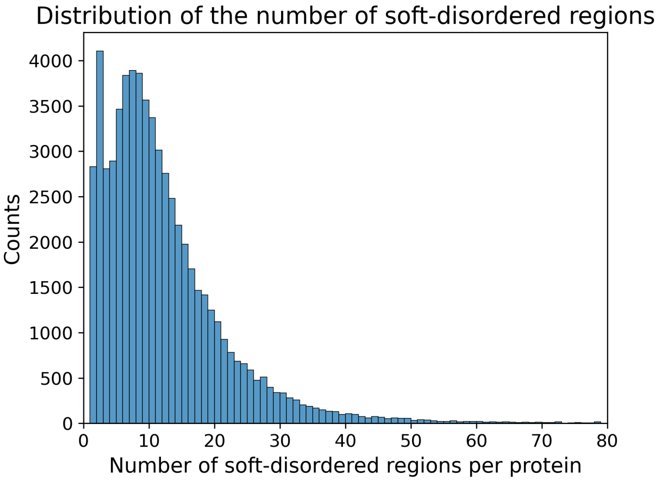


Fig. S2: Distribution of the number of soft-disordered regions per protein in SoftDis clustered dataset.
